# Correlation between spot and 24h proteinuria: Derivation and validation of equation to estimate daily proteinuria

**DOI:** 10.1371/journal.pone.0214614

**Published:** 2019-04-02

**Authors:** Yih-Ting Chen, Heng-Jung Hsu, Cheng-Kai Hsu, Chin-Chan Lee, Kuang-Hung Hsu, Chiao-Yin Sun, Chun-Yu Chen, Yung-Chang Chen, Yi-Ching Yu, I-Wen Wu

**Affiliations:** 1 Department of Nephrology, Chang Gung Memorial Hospital, Keelung, Taiwan; 2 College of Medicine, Chang Gung University, Taoyuan, Taiwan; 3 Healthy Aging Research Center, Laboratory for Epidemiology, Department of Health Care Management, Chang Gung University, Taoyuan, Taiwan; 4 Department of Neurology, Chang Gung Memorial Hospital, Keelung, Taiwan; International University of Health and Welfare, School of Medicine, JAPAN

## Abstract

Daily urine protein (UP) loss is a cumbersome but important measurement to guide diagnosis and treatment of renal disease. Spot urine protein-creatinine ratio (UPCR) can been applied to estimate daily proteinuria. However, the correlations between spot and 24h proteinuria remain controversial. In this cross-sectional study, simultaneous collection of 24h and spot urines were performed from 1,039 (derivation cohort) and 204 CKD patients (validation cohort) of Chang Gung Memorial Hospital, from 2007 to 2017. The correlations between spot UPCR and 24h proteinuria were compared. The mean age of patients of derivation and validation cohort was 63 and 55 years and the mean estimated glomerular filtration rate was 62 ± 35 and 59 ± 36 mL/min/m^2^, respectively. The correlation coefficient was 0.819 between UPCR and 24hUP. Prediction equation was derived as: Log_10_24hUP (g) = 0.814 x Log_10_UPCR (mg/mg) + 0.110 x Gender– 0.004 x Age + 0.004 x Body weight (kg) + 0.002 x CKD stage coefficient– 0.018, where CKD stage coefficient: CKD stage G1 = 1, G2 = 2, G3a = 3.1, G3b = 3.2, G4 = 4, G5 = 5. Correlation coefficient between measured and predicted 24hUP among derivation group and validation group is 0.866 and 0.915, respectively. However, the agreement of spot and daily estimates was less pronounced with proteinuria > 3g than lower values in Bland-Altman analysis. Spot UPCR can accurately predict 24hUP in patients with daily proteinuria below 3g. The development of this equation may facilitate estimation of 24hUP in the clinical practice.

## Introduction

The prevalence of Chronic Kidney Disease (CKD) is increasing worldwide and timely diagnosis is pivotal to preserve renal function [1]. Proteinuria represents an early and essential element for diagnosis, assessment of disease severity and monitoring of treatment response in several renal diseases [2–4]. Estimation of 24h urine protein (UP) has traditionally been considered as the gold standard to determining degree of UP excretion. However, this method is time-consuming, cumbersome for patients and influenced by urine collection process, circadian rhythm and handling methods. Therefore, spot urinary protein/creatinine ratio (UPCR) has been used as substitute for timed UP collection.

Several studies have investigated the correlations between UPCR and urine excretion among different renal disease groups, including lupus nephritis [5], pre-eclampsia [6], glomerular disease [7], kidney transplantation [8] and CKD patients [9–11]. However, the results were inconclusive because of discrepancy on the sample size, severity of proteinuria and renal function, heterogenicity of underlying disease and the procedure of urine collection. Ascertainment of such correlation is mandatory to simplify clinical practice in the management of CKD patients. Furthermore, development of prediction equation to estimate 24hUP was limited by the inconsistent correlation between random and daily sample, biological variation and difference of variable used for model construction [7, 12–14]. Establishment of simple and accurate prediction from spot urine could facilitate clinical assessment of renal patients.

In this study, we assessed the correlation between spot and 24hUP in patients with diverse degree of proteinuria (derivation cohort), developed prediction equation to estimate 24hUP from single spot urine and validated the equation in an independent second cohort.

## Materials and methods

### Patients

**Fig 1:**
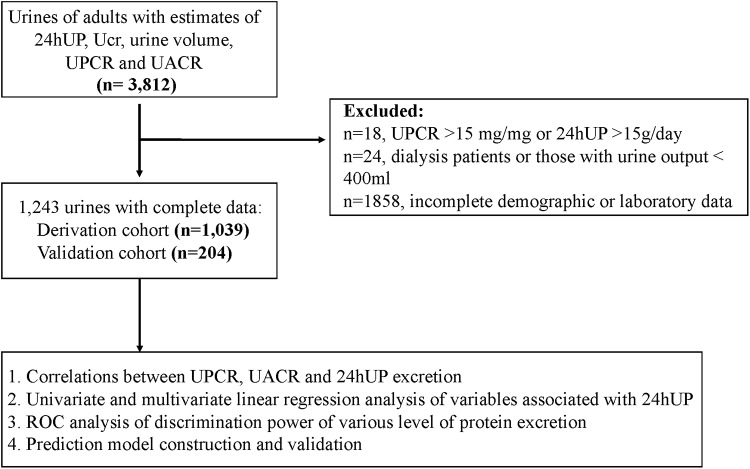
Study flow chart. Abbreviation: 24hUP, 24h urine protein; Ucr, urine creatinine; UPCR, urine protein-creatinine ratio; UACR, urine albumin-creatinine ratio; ROC, operating characteristic curve.

The study enrolled predialysis CKD patients aged greater than 20 years old who had visited nephrology outpatient department of Chang Gung Memorial Hospital at Keelung between 2007 January to 2017 January. Informed written consent were obtained from all these patients. CKD was defined if patients had kidney damage manifested by presence of proteinuria or estimated glomerular filtration rate (eGFR) < 60ml/min/1.73m^2^ (calculated using the 4-variable Modification of Diet in Renal Disease equation) for > 3 months in two separate occasions [15]. Patients having documented fever, urinary tract infection, indwelling urinary catheter, pregnancy and recipients of dialysis therapy or renal transplant graft were excluded from the study. Patients with proteinuria secondary to systemic disease (i.e., systemic lupus erythematous, rheumatoid arthritis, Sjogren’s syndrome, viral infections, syphilis, etc) were excluded because the amount of proteinuria could be largely dependent on the treatments of underlying disease. Those participants with inadequate urine sampling, defined as spot UPCR > 15 mg/mg or 24hUP > 15 g/day (n = 18) and 24h urine amount < 400 mL (n = 24) were also excluded from analysis. Finally, 1,243 patients and 2,486 observations (including morning spot urine and 24h urine sample) were enrolled for data analysis. The derivation cohort included urines of 1,039 patients collected from 2007 to 2015 and validation cohort used urines of participants enrolled from 2016 to 2017 (n = 204, Fig 1). The demographic and clinical information, including age, gender, body weight, height, blood pressure, serum albumin, uric acid, triglyceride, creatinine, the etiology of CKD (diabetes mellitus, hypertension, chronic glomerulonephritis or unknown) and protein intake were accurately recorded at baseline. This study was conducted in accordance with declaration of Helsinki and approved by the ethics committee of the institutional review board at the Chang Gung Memorial Hospital.

### Urine specimens

All the subjects were instructed to start the 24h urine collections after their first voiding in the morning and to collect all urine continuously for 24h, including the last void at the end of the 24h period. The first early morning spot urine sample was collected immediately after the completion of 24h urine collection to estimate spot urine estimates. Both the 24h and spot urine specimen were assayed for volume, UP, albuminuria and urine creatinine (Ucr) concentration. UP concentration was assayed by using the Microprotein Reagent (Beckman Coulter, Fullerton, CA), which is based on a pyrogallol red-molybdate colorimetric method, and the Ucr level, by using the Beckman Synchron creatinine reagent according to the Jaffé kinetic alkaline picrate method. Serum total protein and creatinine concentrations were assayed using Beckman Synchron LX machine.

### Statistics analysis

Descriptive variables were summarized using median (interquartile range), mean (standard deviation), frequencies (percentage) as appropriate. All variables were tested for normal distribution by Kolmogorov-Smirnov test. Student t test was employed to compare means of continuous variables and normally distributed data; otherwise, Mann-Whitney U test was employed. Categorical data were tested by chi-square test or Fisher exact test. Variables related to 24hUP excretion were assessed with univariate linear regression analysis, and variables that were statistically significant (*p*<0.05) in the univariate analysis were included in multivariate analysis by applying a multiple linear regression. We constructed an equation for the 24hUP by employing UPCR and other variables by linear regression from the validation cohort. Variance Inflation Factor (VIF) calculation was performed to address issue of collinearity by using principle component analysis. Validation of this equation was performed in the validation and derivation cohort by correlation coefficient and Bland-Altman analysis. Some variables were missing in minor proportion. Values of serum uric acid were missing in 36 cases (3.5%); body mass index and serum albumin (25, 2.4%), serum triglyceride (24, 2.3%) and body weight (19, 1.8%). The case listwise with missing value were excluded from analysis during linear regression analysis. The correlation between spot UPCR and 24hUP (in grams) was measured by Pearson’s correlation coefficients, and the degree of agreement by Bland-Altman analysis. Receiver-operating characteristic (ROC) analyses were done to assess the diagnostic utility of spot UPCR in predicting 24hUP excretion above specified clinically useful thresholds: >0.15 g, >0.3 g, >0.5 g, >1.0 g, >3.5 g, >4.0 g and >8.0 g. Discrimination was explored using the area under ROC (AUROC). Sensitivity, specificity, positive likelihood ratio and negative likelihood ratio were identified after best Youden’s index was calculated. Accuracy was defined as the proportion of patients correctly identified in the entire ROC analysis. Performance of our prediction has further compared with three previous published equation using derivation cohort [7, 14, 16]. Difference was calculated by measured 24hUP—predicted 24hUP. Bias was presented as the median difference (measured 24hUP—predicted 24hUP) and percent difference [(measured 24hUP—predicted 24hUP) / measured 24hUP) x 100%]. The percentage of predicted 24hUP within 20% (P20), 30% (P30), and 50% (P50) of measured 24hUP and root mean square error were calculated for overall accuracy. All statistical tests were 2-tailed; a *p*-value < 0.05 was considered statistically significant. Data were analyzed using SPSS 21.0 (SPSS inc, Chicago, IL, USA).

## Results

### Baseline characteristics of study population

Table 1 lists the demographic and clinical characteristics of both the derivation (n = 1,039) and validation cohorts (n = 204). The mean age of patients of derivation and validation cohort was 63 ± 14 vs. 65 ± 14 years (p = 0.23). The mean eGFR was 62 ± 35 mL/min/1.73m^2^ vs. 59 ± 36 mL/min/1.73m^2^ (p = 0.27), respectively. Table 2 compares the correlation between spot UPCR and 24hUP. The overall correlation between spot UPCR and 24hUP was 0.819. Among different CKD stages, correlation coefficients between UPCR and 24hUP were larger than 0.80 except for CKD stage G0 (r = 0.180) and stage G3b (r = 0.745). Correlations between spot UPCR and 24hUP were stronger in men than women (r = 0.873 and 0.795, respectively). The correlation between spot UPCR and 24hUP was lower for diabetic than non-diabetic patients (r = 0.798 and 0.883, respectively). In addition, correlation between spot urines with 24hUP were higher in elderly patients than younger ones (r = 0.857 and 0.765, respectively). To avoid individual variation of protein or creatinine excretion secondary to body weight, body surface area (BSA) adjusted 24hUP was used to correlate with spot UPCR. Overall, the correlation coefficients improved by using BSA-adjusted estimates and it was consistent among different subgroups (CKD stage, gender, diabetes mellitus status or age stratum, Table 2).

**Table 1:** Descriptive characteristic of patients in the derivation and validation cohorts.

	Derivation cohort (n = 1,039)	Validation cohort (n = 204)
Age, mean, years	63 ± 14	65 ± 14
Men, number (%)	540 (52)	114 (56)
Body weight, mean, kg	65.2 ± 13.0	64.2 ± 12.7
BMI, mean, kg/m^2^	25.4 ± 4.7	25.4 ± 4.2
Body surface area, mean, m^2^	1.69 ± 0.22	1.67 ± 0.22
Triglyceride, mg/dl	159 ± 217	153 ± 213
Albumin, g/dl	4.3 (4.0, 4.5)	4.2 (3.9, 4.5)
Uric acid, mg/dl	6.6 ± 1.9	6.5 ± 1.8
eGFR, ml/min/1.73m^2^	62 ± 35	59 ± 36
Causes of CKD, number (%)		
Chronic glomerulonephritis	37 (3.6)	7 (3.4)
Diabetes mellitus	340 (32.7)	60 (29.4)
Hypertension	154 (14.8)	27 (13.2)
Urinary tract abnormalities	9 (0.9)	4 (2.0)
Chronic interstitial nephritis	1 (0.1)	0 (0.0)
Congenital kidney disease	6 (0.6)	4 (2.0)
Gout and Hyperuricemia	46 (4.4)	4 (2.0)
Unknown	309 (29.7)	79 (38.7)
Missing data	137 (13.2)	19 (9.3)
CKD stage (GFR)		
Stage G0	29 (2.8)	8 (3.9)
Stage G1	218 (21)	31 (15.2)
Stage G2	269 (25.9)	46 (22.5)
Stage G3a	136 (13.1)	34 (16.7)
Stage G3b	168 (16.2)	38 (18.6)
Stage G4	154 (14.8)	33 (16.2)
Stage G5	65 (6.3)	14 (6.9)
CKD stage (Albuminuria)		
Stage A1	105 (10.1)	13 (6.4)
Stage A2	460 (44.3)	94 (46.1)
Stage A3	474 (45.6)	97 (47.5)
24hUP, g/day	0.26 (0.13, 0.79)	0.27 (0.13, 0.99)
24hUcr, g/day	1.56 (1.02, 2.60)	1.50 (1.10, 2.28)
24h urine amount, ml	1800 (1400, 2400)	1700 (1300, 2050)
Spot UPCR, mg/mg	0.14 (0.08, 0.56)	0.16 (0.08, 0.57)
Calculated protein intake, g/day	58.05 ± 27.14	58.19 ± 20.37

Data were expressed in number (%), mean ± SD or median (25th percentile, 75th percentile).

Abbreviation: BMI, body mass index; CKD, chronic kidney disease; eGFR, estimated glomerular filtration rate; UPCR, urine protein-creatinine ratio.

**Table 2:** Correlations between UPCR and 24hUP (or BSA-adjusted 24hUP) in the derivation group.

	Number	Correlation Coefficient		Interpretation	*p*-value
		Crude	BSA-adjusted		
Overall	1039	0.819	0.839	high	<0.001*
CKD stage					
G0	29	0.180	0.194	negligible	0.350*/0.312†
G1	218	0.840	0.851	high	<0.001*†
G2	269	0.835	0.839	high	<0.001*†
G3a	136	0.793	0.812	high	<0.001*†
G3b	168	0.745	0.787	high	<0.001*†
G4	154	0.825	0.839	high	<0.001*†
G5	65	0.808	0.822	high	<0.001*†
Gender					
Men	540	0.873	0.885	high	<0.001*†
Women	499	0.795	0.810	high	<0.001*†
Diabetes mellitus					
Yes	394	0.798	0.829	high	<0.001*†
No	645	0.883	0.894	high	<0.001*†
Age, years					
≥60	633	0.857	0.876	high	<0.001*†
<60	406	0.765	0.779	high	<0.001*†

CKD, chronic kidney disease; BSA, Body surface area.

* *p*-values, UPCR vs. 24hUP.

^†^ *p*-value, UPCR vs. BSA-adjusted 24hUP.

### Development and validation of prediction equation

Univariate linear regression analysis was followed by multivariate linear regression analysis and has identified the age, gender, body weight and log_**10**_UPCR as independent predictors to estimate 24hUP. A prediction equation best describing the relationship of 24hUP and UPCR was developed from the derivation cohort as: Log_10_24hUP (g) = 0.814 x Log_10_UPCR (mg/mg) + 0.110 x Gender– 0.004 x Age + 0.004 x Body weight (kg) + 0.002 x CKD stage coefficient– 0.018; where the CKD stage coefficients were: CKD stage G1 = 1, G2 = 2, G3a = 3.1, G3b = 3.2, G4 = 4, G5 = 5 (Table 3). The collinearity was checked by using principle component analysis with variables of age, gender and CKD stage. The multiple correlation coefficient was found as 0.166 (with gender) or 0.454 (with age) variable in the model. According to VIF calculation, the value was found as 1.43, less than a preset critical point for potential collinearity problem, VIF>5. Further examination of our presented models, many of the independent variables were chosen from part of these variables which were shown no collinearity problem after the analyses. Therefore, there was no significant effect of collinearity phenomena impacted on the instability of the regression model.

**Table 3:** Multivariate linear regression analysis of variables associated with 24hUP excretion after logarithm transformation.

Variable	Unstandardized coefficient	95% CI		*p*-value
		Lower	Upper	
Age	-0.004	-0.005	-0.002	<0.001
Gender	0.111	0.076	0.144	<0.001
Body weight	0.004	0.003	0.005	<0.001
CKD stage coefficient*	0.002	-0.014	0.019	0.783
Log_10_UPCR	0.814	0.783	0.845	<0.001
Constant	-0.017	-0.137	0.101	0.763

Prediction equation: Log_10_24hUP (g) = 0.814 x Log_10_UPCR (mg/mg) + 0.110 x Gender– 0.004 x Age + 0.004 x Body weight (kg) + 0.002 x CKD stage coefficient– 0.018; Model R^2^ = 0.80; male = 1, female = 0;

*CKD stage coefficient: CKD stage G1 = 1, G2 = 2, G3a = 3.1, G3b = 3.2, G4 = 4, G5 = 5.

Abbreviation: CKD, chronic kidney disease; UPCR, urine protein-creatinine ratio.

**Fig 2:**
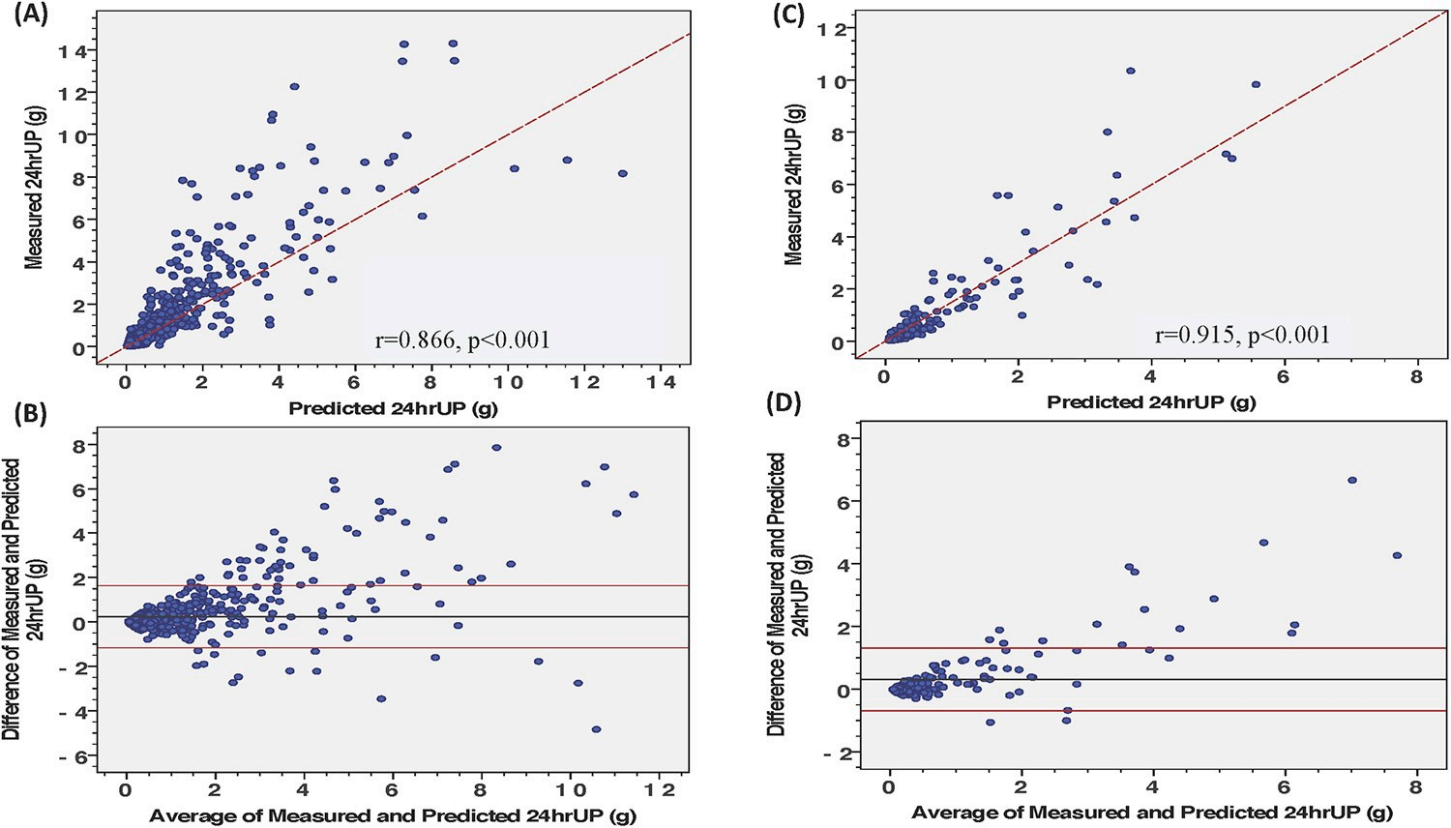
Scatter plot (regression line y = 1.0714x, A) and Bland-Altman analysis (B) comparing predicted with measured 24h urine protein from derivation cohort (n = 1,039). Scatter plot (regression line: y = 1.5x, C) and Bland-Altman analysis (D) comparing predicted with measured 24-hour urine protein from validation cohort (n = 204). The R^2^ of model is 0.80.

The Pearson’s correlation coefficient between measured and predicted 24hUP is 0.866 (R^2^ = 0.80, *p*-value<0.001) in derivation cohort (Fig 2A). Bland-Altman analysis comparing actual and predicted 24hUP from derivation cohort revealed uniform agreement with UP excretion below 3.0g (Fig 2B). Validation of this prediction equation in a second cohort (validation cohort) found a correlation coefficient of 0.915 between predicted and measured estimated (Fig 2C). The Bland-Altman analysis comparing actual and predicted 24hUP of validation cohort illustrated high variability after nephrotic range of proteinuria (>3g/d, Fig 2D). The development and validation of prediction equation to estimated BSA-adjusted 24hUP were described in S1 Table. However, the performance of prediction equation to estimate BSA-adjusted 24hUP from spot UPCR was similar to the original model (R^2^ of the original model is 0.80, and the BSA-adjusted one is 0.79, S1 Fig).

To further clarify the correlation of spot UPCR to 24hUP among situations of different degrees of proteinuria, we stratified urines into daily proteinuria thresholds of <0.15g, 0.3g, 0.5g, 1.0g, 3.5g, 4.0g and 8.0g. A spot UPCR > 0.62 represent threshold to correlate with a 24hUP of > 1g (positive likelihood ratio of 17.18, negative likely ratio of 0.06, accuracy of 0.95 and AUROC of 0.97, Table 4).

**Table 4:** Threshold of spot UPCR to detect different degrees of daily proteinuria in derivation cohort (n = 1,039).

24hUP	>0.15g	>0.3g	>0.5g	>1.0g	>3.5g	>4.0g	>8.0g
Number	727	456	337	218	79	68	22
Spot UPCR threshold	0.17	0.19	0.36	0.62	0.90	1.02	2.48
Sensitivity	0.60	0.82	0.85	0.95	0.98	1.00	1.00
Specificity	0.93	0.88	0.95	0.95	0.88	0.89	0.95
Positive likelihood ratio	8.48	6.97	15.72	17.18	7.68	8.70	19.23
Negative likelihood ratio	0.43	0.21	0.16	0.06	0.03	0.00	0.00
Best Youden index	0.53	0.70	0.80	0.89	0.85	0.89	0.95
Accuracy	0.76	0.85	0.90	0.95	0.92	0.94	0.97
AUROC	0.81	0.91	0.96	0.98	0.97	0.98	0.98

Abbreviation: 24hUP, 24-hours urine protein; UPCR, urine protein-creatinine ratio; AUROC, area under receiver operating characteristic curve.

### Performance of our prediction equation

Table 5 summarized the performance of our prediction equation with three previous published prediction equation using biological estimates of derivation cohort. Correlation coefficient was the highest among all the predictions. The median difference was 12 (IQR: -63, 159) and the median percent difference was 5 (IQR: -35, 40). We observed small bias (5%) in our equation indicating relatively acceptable precision. For accuracy, 25% of patients of our validation cohort found to have a predicted 24hUP within 20% of the measured 24hUP (P20). The 82% of patients of our validation cohort corresponded within 50% of the measured 24hUP. This numbers were remarkable compared with 59% obtained from Ubukata equation [14] or 60% from Hogan equation [7] (Table 5).

**Table 5:** Comparison of new and existing 24hUP excretion estimation equation in the derivation group.

Prediction equation	Correlation coefficient	Difference, mg/day‖	Percent Difference, %¶	Accuracy			RMSE
				P20 (%)	P30 (%)	P50 (%)	
Chen*	0.866	12 (-63, 159)	5 (-35, 40)	25	40	82	1.0034
Teo†	0.847	199 (88, 595)	77 (64, 85)	1	3	10	1.8756
Ubukata‡	0.828	52 (-16, 180)	24 (-8, 54)	27	38	59	1.1541
Hogan§	0.834	53 (-25, 227)	24 (-15, 52)	26	38	60	1.1082

Difference and percent difference were expressed in median (IQR).

* Chen Equation: Log_10_24hUP (g) = 0.814 x Log_10_UPCR (mg/mg) + 0.110 x Gender– 0.004 x Age + 0.004 x Body weight (kg) + 0.002 x CKD stage coefficient– 0.018; CKD stage coefficient: CKD stage G1 = 1, G2 = 2, G3a = 3.1, G3b = 3.2, G4 = 4, G5 = 5.

^†^Teo Equation: Log 24hUP (g) = −0.617019 + 0.7150918 x Log UPCR (mg/mg)

^‡^Ubukata Equation: 24hUP (g) = UPCR x [(Body weight (kg) x 14.89 + Height (cm) x 16.14 –Age x 2.043–2244.45) / 1000)]

^§^Hogan Equation: Log 24hUP (g) = 0.88 x Log UPCR (mg/mg)

^‖^Difference = measured 24hUP—predicted 24hUP (positive indicate measured value > predicted value)

^¶^Percent Difference = (measured 24hUP—predicted 24hUP) / measured 24hUP x 100%

Abbreviation: IQR, Interquartile range; RMSE, root mean square error.

## Discussion

Patients with proteinuria have increased risk for renal progression, cardiovascular disease and mortality. The amount of proteinuria has not only risk prediction significance but it also is useful for monitoring disease activity or progression. The 24h collection of urine represents a cumbersome task for daily practice. Accurate and easy prediction of daily proteinuria from spot urines is mandatory to facilitate clinical management. This study confirmed a good correlation between the UPCR and 24hUP in a CKD cohort and developed an accurate prediction equation to estimate 24hUP from a single spot UPCR. This prediction has further validated in a second cohort showing high agreement. The findings of present study offered a fast and accurate method to simply routine practice for assessment of proteinuria in CKD patients.

Researches investigating surrogate of measurement of 24hUP have shown inconclusive results [10, 17–21]. Strong correlation between UPCR and 24hUP was found in type 1 diabetic patients [17] and pediatric cohort [12]. The accuracy of prediction can be improved by multiplied spot UPCR to estimated Ucr in both proteinuric and non-proteinuric patients [12]. However, Hogan et al. reported moderate correlation between the random UPCR and 24hUP in urine of patients with biopsy-proven glomerular disease. Age, obesity and the logarithmic transformation of urine ratio can influence the correlation between random and daily urine estimates [7]. Price et al. found good correlation of UPCR and 24hUP; however, the cut-off values of significant proteinuria varied significantly among different disease entities [22]. Similarly, Ubukata et al. found a significant correlation between the Up/Ucr and 24hUP in the low (<1.0 g/day) and high urinary protein (>3.5 g/day) groups, whereas the correlation coefficient was lower in the intermediate urinary protein group (1.0–3.5 g/day) [14]. The discrepancy of the prediction between different studies may be attributed to several factors, including different ethnic group, age, muscle size, circadian rhythm of urine collection or dietary pattern [23, 24]. The prediction of 24hUP from spot UP was strong across different subgroups of our patient cohort (gender, age, presence of diabetes or different CKD stage, Table 2). The high accuracy of prediction was observed among patients with different degrees of proteinuria (Table 4) in two independent cohorts. The protein intake did not differ between the two cohorts. In spite of the robustness of our model, further large-scale validation should be needed to establish universal consensus.

Age, gender, body weight, Ucr and race are common variables used to estimate 24hUP [7, 12–14, 25]. In this study, we integrated several important demographic factors including age, gender, body weight and CKD stage, in addition to the spot UPCR for estimation of 24hUP. The correlation of estimated and measured 24hUP using our equation was higher than those of these studies. The age, gender, and body weight actually reflect the muscle size of patient and, in turn, may contribute to the amount of Ucr concentration. The CKD stage may in part influence the degree of proteinuria of disease. Peculiarly, despite of overall high correlations between spot UPCR and 24hUP among different stages of CKD, we observed a slightly lower correlation between spot UPCR and 24hUP in patients of CKD stage G3b (r = 0.745, *p*<0.001) compared with patients of other stages. The relative wide range of proteinuria and high variability of patients due to diverse disease etiology may contribute to this dissimilarity between patients of different CKD stages.

Nephrotic-range proteinuria represents a critical clinical condition that warrants further diagnosis or treatment to preserve renal function. Rapid ascertainment of such degree of proteinuria may guide medical decision. Renal biopsies should mandatory recommended to confirm renal damage. On the other hand, daily proteinuria of 1g serves as indicator of prognosis or monitor of treatment response in glomerular disease. By using our prediction equation, spot UPCR of 0.9 in a CKD stage G1 man of 60 years and 70 kg corresponded to a daily proteinuria of 1.25g; in a CKD stage G3a woman with similar age and anthropometry may have a daily proteinuria of 979 mg. This may represent a practical way to prompt medical decisions.

Here, we reported significant correlation between estimated 24hUP by using spot UPCR and the daily collected proteinuria. However, several limitations of the study should be addressed. First, the estimation has been developed from single ethnic population which limited the generalizability of study. Second, the information on the drug usage in these patients were unknown. Some antiproteinuric drugs, such as those drugs interfering with renin-angiotensinogen-aldosterone system or pentoxifylline, may influence on the protein excretion of participants. Finally, equation-based prediction of 24hUP in patients with severe proteinuria should be interpreted with caution. In spite of high accuracy to predict 24hUP using spot urine in patients with heavy proteinuria (Table 4), the affirmation may be limited by the relatively scarce number of patients with heavy proteinuria in our derivation cohort. In addition, correlations between predicted and measured value were more consistent for urines with proteinuria below 3g than higher levels of proteinuria among both derivation and validation groups (Fig 2B and 2D). Therefore, 24h urine sample collection should still be needed for proteinuria quantification in patients with severe proteinuria. However, the use of two independent cohorts with large sample size and wide range of proteinuria across diverse disease etiology, establishment of standard protocol for urine collection which ensure accuracy of urine preparation, evaluation between same day morning-spot urine and 24h urine collections which can limit intra-individual and inter-day protein excretion variability [26–28] and the comparison of performance among different prediction equations may strengthen the conjecture of our supposition. Further prospective study should need to validate our finding in different populations.

In conclusion, spot UPCR can accurately predict 24hUP in patients with daily proteinuria below 3g. The development of a simple equation: Log_10_24hUP (g) = 0.814 x Log_10_UPCR (mg/mg) + 0.110 x Gender– 0.004 x Age + 0.004 x Body weight (kg) + 0.002 x CKD stage coefficient– 0.018, may facilitate clinical practice for estimation of daily proteinuria. The exact role of this prediction equation in long-term patient outcome remains to be elucidated.

## Supporting information

S1 Table Multivariate linear regression analysis of variables associated with BSA-adjusted 24hUP excretion after logarithm transformation. Prediction equation: Log_10_24hUP (g) = 0.813 x Log_10_UPCR (mg/mg) + 0.104 x Gender– 0.004 x Age + 0.003 x CKD stage coefficient + 0.256; Model R^2^ = 0.79; male = 1, female = 0; *CKD stage coefficient: CKD stage G1 = 1, G2 = 2, G3a = 3.1, G3b = 3.2, G4 = 4, G5 = 5. Abbreviation: CKD, chronic kidney disease; UPCR, urine protein-creatinine ratio. BSA, body surface area.
(DOCX)

S1 Fig Scatter plot (regression line y = 1.25x, A) and Bland-Altman analysis (B) comparing predicted with measured BSA-adjusted 24h urine protein from derivation cohort (n = 1,039). Scatter plot (regression line: y = 2x, C) and Bland-Altman analysis (D) comparing predicted with measured BSA-adjusted 24-hour urine protein from validation cohort (n = 204). The R^2^ of model is 0.79.
(TIFF)

S1 Dataset Raw data used for this manuscript. (XLSX)
