# Supplementary material for: Correlation between spot and 24h proteinuria: Derivation and validation of equation to estimate daily proteinuria
Source: PLoS One. 2019 Apr 2;14(4):e0214614. doi: 10.1371/journal.pone.0214614 (PMC6445407; doi:10.1371/journal.pone.0214614)
Supplement: S1 Table — Prediction equation: Log1024hUP (g) = 0.813 x Log10UPCR (mg/mg) + 0.104 x Gender– 0.004 x Age + 0.003 x CKD stage coefficient + 0.256; Model R2 = 0.79; male = 1, female = 0; *CKD stage coefficient: CKD stage G1 = 1, G2 = 2, G3a = 3.1, G3b = 3.2, G4 = 4, G5 = 5. Abbreviation: CKD, chronic kidney disease; UPCR, urine protein-creatinine ratio. BSA, body surface area. (DOCX) [file pone.0214614.s001.docx]

**S1 Table. Multivariate linear regression analysis of variables associated with BSA-adjusted 24hUP excretion after logarithm transformation**

| **Variable** | **Unstandardized coefficient** | **95% CI** | | | ***p-*value** |
| --- | --- | --- | --- | --- | --- |
|  |  | **Lower** | **Upper** | |  |
| Age | -0.004 | -0.005 | | -0.002 | <0.001 |
| Gender | 0.104 | 0.072 | | 0.135 | <0.001 |
| CKD stage coefficient^*^ | 0.003 | -0.013 | | 0.020 | 0.688 |
| Log_10_UPCR | 0.813 | 0.782 | | 0.844 | <0.001 |
| Constant | 0.256 | -0.137 | | 0.101 | <0.001 |

Prediction equation: Log_10_24hUP (g) = 0.813 x Log_10_UPCR (mg/mg) + 0.104 x Gender – 0.004 x Age + 0.003 x CKD stage coefficient + 0.256; Model R^2^=0.79; male=1, female=0; *CKD stage coefficient: CKD stage G1=1, G2=2, G3a=3.1, G3b=3.2, G4=4, G5=5.

Abbreviation: CKD, chronic kidney disease; UPCR, urine protein-creatinine ratio. BSA, body

surface area
