# Supplementary figures and images for: Correlation between spot and 24h proteinuria: Derivation and validation of equation to estimate daily proteinuria
Source: PLoS One. 2019 Apr 2;14(4):e0214614. doi: 10.1371/journal.pone.0214614 (PMC6445407; doi:10.1371/journal.pone.0214614)

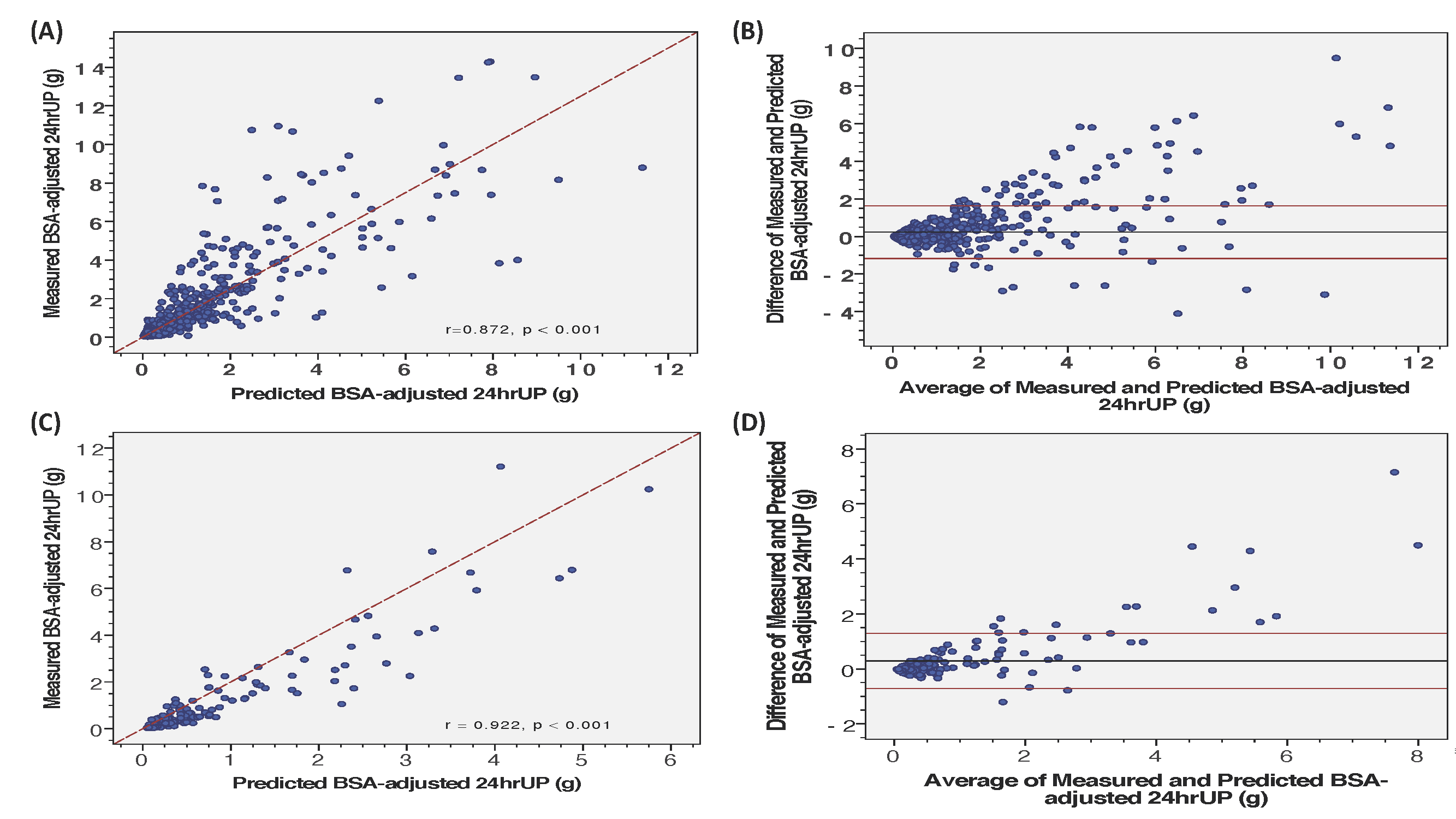

Supplement: S1 Fig — Scatter plot (regression line y = 1.25x, A) and Bland-Altman analysis (B) comparing predicted with measured BSA-adjusted 24h urine protein from derivation cohort (n = 1,039). Scatter plot (regression line: y = 2x, C) and Bland-Altman analysis (D) comparing predicted with measured BSA-adjusted 24-hour urine protein from validation cohort (n = 204). The R2 of model is 0.79. (TIFF) [file pone.0214614.s002.tiff]
